# The Role of Radiotherapy for Patients With Multiple Myeloma in the Modern Era: A Real World Single‐Centre Experience

**DOI:** 10.1002/jha2.70141

**Published:** 2025-10-04

**Authors:** Dipal Mehta, Maria Gabriel, Nathan Adu‐Poku, Nikolaos Kanellias, Xenofon Papanikolaou, Jonathan Sive, Neil Rabin, Rakesh Popat, Eileen M. Boyle, Lydia Lee, Kwee Yong, Agapi Parcharidou, Suganya Sivabalasingham, Karimali Keshwani, Ke Xu, Charalampia Kyriakou

**Affiliations:** ^1^ Department of Haematology University College Hospitals London NHS Foundation Trust London UK; ^2^ Clinical Therapeutics University of Athens Alexandra Hospital Athens Greece; ^3^ Northwick Park Hospital London North West University Healthcare NHS Trust London UK; ^4^ Department of Clinical Oncology University College Hospitals London NHS Foundation Trust London UK

**Keywords:** myeloma, myeloma treatment, radiotherapy

## Abstract

**Background:**

Radiotherapy (RT) is a well‐established treatment modality in multiple myeloma (MM), particularly for skeletal‐related events. However, its role is likely evolving with the introduction of modern systemic treatments.

**Methods:**

We conducted a retrospective analysis of a large, single‐centre MM cohort treated with contemporary protocols to describe current patterns of RT use.

**Results:**

RT continues to offer clinical benefit, with shifting patterns of use observed compared to historical cohorts.

**Conclusion:**

RT remains relevant in the modern MM landscape and may hold increasing value in later treatment stages. We postulate a future role as bridging therapy prior to CAR‐T/bispecific antibody treatment.

**Trial Registration:**

The authors have confirmed clinical trial registration is not needed for this submission

1

Multiple myeloma (MM) is characterised by a proliferation of malignant plasma cells in the bone marrow (BM). Multifocal osteolytic bone disease, alongside bone and soft‐tissue plasmacytomas are hallmarks of MM and represent a major cause of morbidity, leading to pathological fractures, spinal cord compression and severe bone pain [1]. Radiation therapy (RT) has been regarded as an important pillar of MM management owing to the radiosensitive nature of the disease [2]. RT has shown high efficacy in palliating bony pain and has a positive impact on bone recalcification, therefore reducing skeletal‐related events (SREs) and improving quality of life [3, 4].

Previous studies on historical MM cohorts have shown high incidence of patients requiring RT during their disease course, varying from 70% in oldest reported cohorts to 34% in more recent reports [4, 5, 6]. Across these studies, the predominant indication for RT was palliation of bony pain [5, 6, 7]. Common alternative indications included prevention/treatment of pathological fractures, spinal cord compression and management of extramedullary disease (EMD). Overall, RT was associated with excellent pain response, with Matushek et al. showing superior pain relief as well as improved bony recalcification in patients receiving higher RT doses [5].

Advances in MM treatment with the introduction of proteasome inhibitors, immunomodulatory drugs, anti‐CD38 monoclonal antibodies, and more recently, bispecific antibodies and CAR‐T cell therapies, have led to significantly deeper and more durable treatment responses translating to improved survival. Whilst such novel therapies have transformed the MM treatment paradigm, it is likely that the role of RT within this paradigm has also changed significantly. However, in the context of such contemporary treatment protocols, there is limited, recently published data on the use of RT in MM. Particular issues of concern include the optimal timing and dose of RT, the risk of recurrence at the initial radiation site and the effect of RT on peripheral blood stem cell harvest for ASCT. In 2015, Talamo et al. analysed 149 MM patients receiving RT, showing that the majority of RT episodes were administered early in the treatment pathway at the time of initial diagnosis [6]. Toxicity, particularly the development of secondary malignancy, is another important consideration for overall treatment decisions, especially in the current era of advanced therapies that can prolong survival. Here, we retrospectively analysed the use of RT in a large real‐world single‐centre cohort of MM patients.

We used electronic patient records to retrospectively identify patients diagnosed with MM who received RT at University College London Hospital (UCLH) between 1 January 2016 and 31 December 2022. Sites of disease were identified by PET or MRI, as per IMWG criteria. We used GraphPad Prism v10 for statistical analysis, applying the Mann–Whitney *U* test. *p* Values < 0.05 were deemed to be significant.

Out of 906 MM patients who were actively followed up within our institution in the timeframe specified, we identified 157 patients (17%) who had received RT during their disease course. Baseline demographics, disease stage, cytogenetics and systemic treatment details of patients receiving RT are shown in Table 1. The median age at diagnosis was 59 years (range 28–94), with 62% male and 51% White British. The median number of treatment lines was 4 (range 1–9). The majority of patients had previous exposure to bortezomib (91%; 137/151) and lenalidomide (84%; 127/151), with minorities being exposed to pomalidomide (48%; 72/151) and anti‐CD38 monoclonal antibodies (48%; 72/151). Of note, 13% (19/151) had received belantamab and 3% (4/151) CAR‐T/bispecific antibodies. A total of 77% of patients underwent ASCT.

**TABLE 1:** Baseline characteristics and details of systemic treatment.

Demographics	Total = 157 patients
Median age at diagnosis in years (range)	59 (28–94)
Male (%)	98 (62.4%)
Ethnic Origin White British (%)	80 (51.0%)

ISS	
Stage I	53 (33.8%)
Stage II	29 (18.5%)
Stage III	20 (12.7%)
Unknown	55 (35.0%)

Cytogenetic risk	
Standard risk	67 (42.7%)
^a^High risk	28 (17.8%)
Unknown	62 (39.5%)

Type of measurable disease	
IgG	85 (54.1%)
IgA	29 (18.5%)
Light chain only	35 (22.3%)
Other	8 (5.1%)
Unknown	3 (1.9%)

Systemic treatment	Total = 151 patients
Median no. of treatment lines (range)	4 (1–9)
Proteosome inhibitor	
Bortezomib	137 (90.7%)
Carfilzomib	31 (20.5%)
Ixazomib	46 (30.5%)
IMID	
Thalidomide	87 (57.6%)
Lenalidomide	127 (84.1%)
Pomalidomide	72 (47.7%)
Panabinostat	25 (16.6%)
Anti‐CD38 mAb	
Daratumumab	52 (34.4%)
Isatuximab	20 (13.2%)
Belantamab	19 (12.6%)
Bispecific antibody	2 (1.3%)
CAR‐T	2 (1.3%)
Other	15 (9.9%)
Autologous stem cell transplant	116 (76.8%)
Autologous stem cell transplant × 2	22 (14.6%)

^a^ High‐risk cytogenetics defined as presence of t(4;14)/t(14;16) and/or del(17p).

Over a median follow‐up of 71 months (range 3–237 months), we recorded 331 sites of RT across the cohort, with a median of 2 RT episodes per patient (range 1–9). RT details are summarised in Table 2. The most commonly irradiated anatomical sites were the spine (40%; 131/331) and pelvis/sacrum (15%; 48/331). The type of tissue irradiated was predominantly bony (67%; 222/331), followed by bony/paramedullary (22%; 73/331) and extramedullary (11%, 36/331). Indications for RT included palliation of bone pain (66%; 219/331), spinal cord compression (16%; 54/331), post‐surgical consolidation (6%; 21/331), extramedullary disease requiring mass reduction (6%; 19/331) and pathological fracture/prophylaxis of pathological fracture (5%; 18/331). Commonly used RT dosing schedules included 20 Gy delivered over five fractions (51%; 168/331) and 8 Gy delivered over one fraction (34%; 112/331). Across 331 RT sites, the median RT dose was 20 Gy (range 6–45 Gy), and the median number of fractions was 5.14% (46/331) of RT sites had evidence of subsequent site‐specific disease relapse, whilst 1% (3/331) of RT sites were refractory to RT. The mean time from initial RT delivery to same‐site relapse was 31.6 months (range 1.4–174 months). A total of 7% (22/331) of RT sites were re‐irradiated following same‐site relapse.

**TABLE 2:** Details of radiation therapy (RT).

Type of tissue irradiated	Total = 331 RT sites
Bony	222 (67.1%)
Bony/paramedullary	73 (22.1%)
Extramedullary	36 (10.9%)

Anatomical site	
Spine	131 (39.6%)
Femur/tibia	27 (8.2%)
Humerus/radius	25 (7.6%)
Pelvis/sacrum	48 (14.5%)
Skull/facial bones	27 (8.2%)
Ribs/sternum	22 (6.6%)
Shoulder/scapula	17 (5.1%)
Other/soft tissue	28 (8.5%)

Indication for RT	
Palliation of bone pain	219 (66.2%)
Cord compression	54 (16.3%)
Post‐surgical	21 (6.3%)
Pathological fracture/prophylaxis of pathological fracture	18 (5.4%)
Extramedullary disease/mass reduction	19 (5.7%)

Radiation regimen	
5 × 4 Gy	168 (50.8%)
1 × 8 Gy	112 (33.8%)
10 × 3 Gy	21 (6.3%)
4 × 4 Gy	5 (3.2%)
25 × 1.8 Gy	4 (1.2%)
Other	8 (2.4%)
Unknown	13 (3.9%)

Timing of first RT episode (from initial MM diagnosis)	Total = 157 patients
0–3 months	42 (26.8%)
3–12 months	29 (18.5%)
1–2 years	18 (11.5%)
2+ years	59 (37.6%)
Unknown	9 (5.7%)

A total of 27% (42/157) of patients received their first episode of RT within the first 3 months of initial MM presentation. The remainder received initial RT at subsequent time points, including 19% (29/157) at 3–12 months, 12% (18/157) at 1–2 years and 38% (59/157) beyond 2 years. Overall, RT was well tolerated, with few cases of documented short‐term toxicity. A total of 2.5% (4/157) of patients reported localised skin toxicity, and 3% (5/157) reported gut disturbance. A total of 7% (11/157) developed second malignancy, including genitourinary (3/157), colorectal (2/157), lung (1/157), breast (1/157), melanoma (1/157), thyroid (1/157), cancer of unknown primary (1/157) and therapy‐related myelodysplastic syndrome (MDS) (1/157). There was no significant difference in median total RT dose delivered to those who developed second malignancy compared to those who did not (Mann–Whitney *U*; 40 vs. 28 Gy, *p* = 0.62).

With regards to peripheral blood CD34+ stem cell yields for ASCT, whilst there was a trend to greater yield in patients who had not received RT before stem cell harvest, this was not statistically significant (Mann–Whitney *U*; 4.5 × 10^6^/kg vs. 3.9 × 10^6^/kg, *p* = 0.15). However, when comparing patients who had specifically received prior pelvic/lumbosacral RT to those who had not, the difference was significant (Mann–Whitney *U*; 3.8 × 10^6^/kg vs. 5.0 × 10^6^/kg, *p* = 0.023).

Our study involving patients on contemporary treatment protocols showed that RT was used in a relatively small proportion of the overall MM cohort (17%; 157/906), which is less than reported in historical populations [2, 5, 6]. When used, it was mainly at later therapy lines rather than at initial diagnosis, which again differs from previously described cohorts [5, 6]. As with previous studies, the main indication for RT in our cohort was palliation of bony pain. As this was a retrospective study, we did not elucidate the effectiveness of RT in improving pain, although historical reports suggest high efficacy rates of 75%–100% even with short courses [6]. A total of 16% of RT episodes were administered for spinal cord compression, with all cases associated with prevention of long‐term neurodisability. This highlights the invaluable role for RT in managing this acute presentation, thus negating the need for invasive neurosurgical intervention [8].

Whilst RT appeared to be generally well tolerated, the prolonged median follow‐up period of 71 months allowed the identification of longer‐term consequences of RT. We identified an irradiation site relapse rate of 14%. Whilst this is significant, it must be acknowledged that the primary goal in the majority of instances (66%) was pain control rather than disease control. Nonetheless, this reinforces the need for continued long‐term vigilance including at sites of previous RT, as same‐site relapse is not uncommon despite the radiosensitive nature of the disease.

We also found a rate of second primary malignancy of 7% amongst our RT‐treated cohort. It is difficult to prove causality especially in patients who have received multiple lines of systemic therapy, however secondary malignancy is a well‐described late side‐effect of RT [9], and we believe this is highly relevant amidst the improving survival rates in MM patients. Prospective studies comparing rates of second primary malignancy in RT‐treated and RT‐naïve MM patients will be particularly informative to evaluate long‐term consequences in the context of contemporary treatments. Our study also demonstrated a negative impact of pelvic/lumbosacral RT on peripheral blood stem cell yield; this has been described previously [10], and suggests the need to adapt method of stem cell collection to account for this effect, or to reserve RT for post‐harvest and later treatment lines. In addition, we did not observe any abscopal effects of RT related to the concomitant use of immune therapies. Future prospective studies to ascertain whether an abscopal effect exists in this context will be useful.

This single‐centre retrospective study demonstrates that RT continues to hold a role in the era of novel agents for specific acute indications in combination with systemic MM treatment. However, with the increasing availability and success of novel treatments, the specific place for RT in the MM treatment paradigm has likely evolved. Our findings suggest a shift towards the use of RT at later time points, which could potentially prove useful as bridging therapy prior to CAR‐T and bispecific antibody therapies as in the case of the lymphomas, calling for prospective studies to evaluate the efficacy of RT in this setting. This would minimise the long‐term sequelae which will likely become more apparent with improving survival rates. More focused studies on the timing and utility of RT in current treatment pathways are warranted to maximise its use.

## Author Contributions

D.M., S.S., K.X. and C.K. wrote the paper. D.M., M.G., N.A.P. and N.K. collected and analysed data. X.P., N.R., J.S., R.P., E.B., K.Y., L.L., K.K. and A.P. critically reviewed the paper.

## Conflicts of Interest

The authors declare no conflicts of interest.

## Ethics Statement

The authors have nothing to report.

## Data Availability

The datasets generated and/or analysed during the current study are available from the corresponding author on reasonable request.
